# 

**Published:** 1915-10-09

**Authors:** 


### CONTENTS.

PAGE

**The Supply of Medical Students** ... ... ... ... 21

**Something New in Medicine** ... ... ... 22

**Hospital and Institutional News** ... ... ... ... 23

The Poor Babies of Lambeth—Decrease in Infirmary Patients—To Help the British Wounded—A New Superintendent at the Park Hospital—Manchester's Burden—New Zealand Practitioners and Military Service—Winchmore Hill Sanatorium Changes—Austria's Army of Crippled Soldiers—A Charming First Number—Hospital Farming in London—Able Appeals Drafting—Hospitals and the Spirit Tax—The B.M.A. and the Voluntary Hospitals—"An Unfair Burden"—The Proper Function of a Sanatorium—The Winter Session—The Curative Value of Gunpowder—Gunpowder, Shepherds, and Parsons—The Manchester Election—Mr. Frank G. Hazell—Mothers' Rights and Guardians' Wrongs—The Trade Defence of Adulteration—This Week's Drug Market.

**The Regimental Medical Officer** ... ... ... 29

An Outline of His Duties.

**A Medical Causerie** ... ... ... ... ... 31

**Round the World** ... ... ... ... ... 32

Fellow-Workers and the Roll of Honour.

**American Ideals and Hospital Efficiency** ... 33

Looking Forward.

PAGE

**The Work of the R.A.M.C.** ... ... ... ... 34

Another Record of Gallant Deeds.

**National Insurance Act Developments** ... ... 35

The Settlement of Disputes.

**The Department of Modern Nursing** ... ... 36

Samaritan Free Hospital for Women.

**Mothercraft and Child-Welfare Centres** ... 37

(1) How to Manage Schools for Mothers.

**Legal Notes for Institutional Workers** ... 38

Parents and the Mental Deficiency Act—Charitable Bequests to Branch Institutions—Misconduct of Midwives—A Medical Opinion.

**Parliament and the Profession** ... ... ... 39

**The Hospitals of India and Dublin** ... ... 40

The Hospital System of India.

Uniform Compliments Invite Criticism on Dublin Hospitals.

**The Editor's Letter-Box** ... ... ... ... iv

"How Doctors are Wasted."

**Passing Topics of the Day** ... ... ... ... iv

**The Institutional Worker** ... ... (Suppl.) 1

Work for Consumptives in Sanatoria.

Appeals for War Comforts.

Question for October.

Enquiries and Answers.

The Sick and In Need.

Employment and Training.

